# Examining the Effects of the Protection Motivation Theory–Based Online Intervention on Improving the Cognitive Behavioral Outcomes of Caregivers of Children With Atopic Diseases: Quasi-Experimental Study

**DOI:** 10.2196/72925

**Published:** 2025-05-13

**Authors:** Dequan Shen, Qinzhun Zhang, Jiayu Tang, Jiahui Wu, Hui Huang, Yuchang Xu, Yinan He, Jialu He, Chengyin Ye

**Affiliations:** 1 Department of Health Management School of Public Health Hangzhou Normal University Hangzhou China; 2 Department of Clinical Medicine School of Medicine Hangzhou City University Hangzhou China; 3 Department of Epidemiology and Biostatistics School of Public Health Hangzhou Normal University Hangzhou China

**Keywords:** cognitive behavioral therapy, internet-based intervention, protective motivation theory, caregivers, child, hypersensitivity, allergy and immunology, prevention and control

## Abstract

**Background:**

The increasing prevalence of pediatric atopic diseases in China poses substantial risks to children’s physical health, mental well-being, and quality of life. Cognitive behavioral interventions for caregivers are effective in managing pediatric atopic diseases. Existing interventions are typically siloed and lack integration across the comorbidities of the atopic march. The protection motivation theory (PMT) could provide an integrated cognitive behavioral intervention framework for addressing shared pathophysiological mechanisms and unifying management strategies across atopic diseases, while online interventions offer advantages in accessibility, cost-effectiveness, and scalability, particularly for caregiver-mediated pediatric care.

**Objective:**

This study aimed to develop and evaluate a PMT-based cognitive behavioral online (PMT-CBO) intervention for caregivers of children with atopic diseases, assessing its effects on caregivers’ protective motivation, behavioral intentions, preventive practices, and children’s atopic disease outcomes.

**Methods:**

A quasi-experimental design was conducted in 3 health care institutions in Hangzhou, China, where 2 health care institutions were assigned to the PMT-CBO group (127/243, 52.3%) and 1 health care institution was assigned to the control group (116/243, 47.7%). Caregivers in the PMT-CBO group received a 4-week structured course comprising 16 online modules delivered via a WeChat mini-program, whereas controls received routine care with verbal education. Primary outcomes included caregivers’ PMT dimensions (threat appraisal and coping appraisal), behavioral intentions, and preventive behaviors, and secondary outcomes involved children’s symptom severity and medication adherence. The primary outcome scales or questionnaires were designed by the research team, while the secondary outcome scales were derived from established studies. All scales demonstrated good reliability and validity. Intention-to-treat analysis was used.

**Results:**

Compared to the control group, the PMT-CBO group demonstrated significant improvements in overall PMT scores (*Z*=–6.289; *P*<.001) and most subdimensions (response efficacy, self-efficacy, threat severity, and response cost, with *P*<.05), except susceptibility (*Z*=–1.321; *P*=.19) and reward appraisals (*Z*=–0.989; *P*=.32). In the intervention group, caregivers exhibited stronger intentions and partial behavioral optimization (eg, environmental allergen control, with *Z*=–3.025; *P*=.002) and children showed improved medication adherence (*Z*=–4.457; *P*<.001) and alleviated eczema (*Z*=–3.112; *P*=.002) and allergic rhinitis symptoms (*Z*=–3.277; *P*<.001), although no significant differences emerged in asthma control (*Z*=–.830; *P*=.41) or food allergy–related caregiver burden (*Z*=–1.693; *P*=.09).

**Conclusions:**

The PMT-CBO intervention enhanced caregivers’ motivation and intentions and children’s medication adherence and eczema and rhinitis outcomes, with a 91.3% (116/127) completion rate via WeChat’s scalable platform. Limited improvements in asthma control and food allergy management implied the future need for additional condition-specific plug-ins, beyond the core PMT-CBO modules. Moreover, merging this PMT-CBO intervention with implementation techniques or ecological frameworks could help address intention-behavior gaps and external barriers, thereby promoting equitable and precision-based allergy care.

## Introduction

### Background

Atopic diseases, also referred to as the atopic march, describe the progression of allergic conditions during infancy and childhood [1]. This progression typically starts with atopic dermatitis or eczema and subsequently progresses to immunoglobulin E–mediated food allergies and allergic rhinitis, eventually leading to asthma if not effectively managed [2,3]. Once initiated, it becomes challenging to halt its course. Epidemiological studies indicate that approximately 75% of children with atopic dermatitis will develop allergic rhinitis, and more than 50% will progress to asthma [1]. In China, the incidence and prevalence of these diseases are showing a rapid upward trend [4]. The prevalence of atopic dermatitis among children aged 1 to 7 years in China was 13% [5]. The overall prevalence of self-reported food allergies among Chinese children aged 0 to 5 years was 4.81% [6]. A meta-analysis estimated that the overall prevalence of allergic rhinitis in Chinese children was 22% [7]. The prevalence of “ever asthma” and physician-diagnosed asthma (16% and 5.3%, respectively) in children aged 3 to 6 years in Shanghai has increased 2-fold over the past 3 decades [8]. The onset and exacerbation of atopic diseases and allergic conditions in children can adversely impact their physical development [9,10], mental health [11], cognitive development [12], and quality of life [13].

Beyond clinical visits and medical treatment, comprehensive caregiver and child education coupled with behavior modification strategies are pivotal for effective atopic disease management. Multidisciplinary interventions have been developed for individual atopic conditions. Among them, some cognitive behavioral therapy approaches targeted at caregiver psychological distress (eg, anxiety, depression, and risk perceptions in food allergy [14-16], asthma [17], and allergic rhinitis [18]), while other educational programs enhanced children’s quality of life and clinical outcomes (eg, reduced disease severity and emergency visits) through caregiver self-efficacy improvement [17,19-21]. However, most existing interventions remain siloed to one specific allergic condition, with limited integration of the atopic march framework despite epidemiological evidence highlighting the high prevalence of comorbid allergic conditions in pediatric populations [22-24]. Given shared pathophysiological mechanisms and management strategies across atopic diseases—including allergen avoidance (eg, dust mite–proof bedding); pharmacotherapy (corticosteroids and antihistamines); barrier protection (nasal saline irrigation and skin moisturizers); immunotherapy; cross-reactivity education; and emergency preparedness (epinephrine autoinjectors) [25,26]—our study posits that unifying these approaches through theory-driven behavioral interventions may synergistically address overlapping pathways. By leveraging the behavior change theory, such integrated strategies could optimize both caregiver and child outcomes while streamlining clinical care for multifactorial allergic conditions.

**Figure 1:**
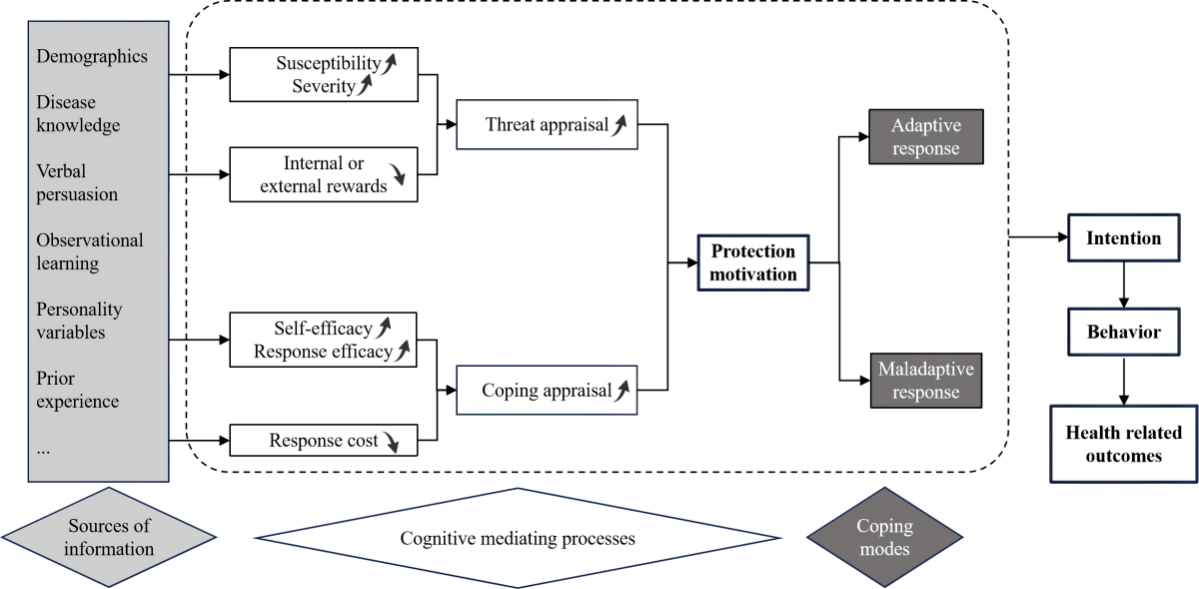
The structure of the protection motivation theory.

The protection motivation theory (PMT) was proposed by Rogers in 1975. Since then, the model has been adopted widely to understand, predict, and modify protective behaviors, particularly health-related behaviors [27]. Figure 1 illustrates the structure of PMT. In this model, information from environmental and intrapersonal sources serves as input. The PMT model itself consists of 2 main elements: cognitive mediators and coping models. The cognitive mediators include 6 dimensions and are further divided into 2 categories: threat appraisal and coping appraisal [28]. Threat appraisal involves evaluating unhealthy behaviors or diseases and includes 3 dimensions: susceptibility (how susceptible an individual feels to the threat), severity (how serious the threat is perceived to be), and internal or external rewards (the benefits of not taking protective action) [27,29]. Coping appraisal involves assessing health behaviors and includes 3 dimensions: self-efficacy (confidence in one’s ability to carry out the adaptive response); response efficacy (belief in the effectiveness of the adaptive response); and response cost (the perceived costs associated with taking the adaptive response, such as time, effort, or money) [30]. Coping modes, on the other hand, represent the behavioral outcomes and can be either adaptive responses (intentions to adopt healthy behaviors) or maladaptive responses (intentions to adopt unhealthy behaviors) [29]. These 6 dimensions within the 2 cognitive mediators form the core constructs of PMT.

Over the past decades, the PMT has been widely applied across diverse health domains with notable efficacy. Its applications extend beyond adult health behavior modification, such as skin cancer prevention [31], prenatal environmental risk education [32], and lifestyle management in military personnel with type 2 diabetes [33]. The PMT has also been effectively used in caregiver-mediated interventions for pediatric populations. These include improving children’s asthma symptoms [34]; oral health [35]; enhancing treatment adherence in muscular dystrophy [36]; promoting COVID-19 vaccination uptake [37]; and mitigating risks related to sexual abuse [38], pornography exposure [39], online hazards [40], and secondhand smoke [41]. Given the PMT’s validated effectiveness in child health management across these applications, we propose it as a theoretically grounded framework for guiding our online cognitive behavioral intervention for pediatric atopic diseases.

Moreover, online health interventions demonstrate advantages over traditional in-person approaches, including enhanced convenience, accessibility, and cost-effectiveness and reduced social stigma through anonymity [42-45]. These online programs have been successfully applied in caregiver-mediated pediatric interventions, spanning unintentional injury prevention [46], breastfeeding support for preterm infants [47], and cancer symptom management [48] as well as applications targeting pediatric atopic diseases (eg, reducing disease severity [19], improving treatment adherence [49,50], and alleviating caregiver psychological distress [18]). These findings collectively highlight the potential of digital behavioral interventions to address multidimensional challenges in atopic disease management.

### Objectives

The primary aim of this study was to conduct a quasi-experimental study to evaluate the effects of a 4-week PMT-based cognitive behavioral online (PMT-CBO intervention) in improving caregivers’ engagement in managing their children’s atopic diseases. Specifically, this study sought to determine the extent to which the intervention improves caregivers’ protection motivation, intentions, and behaviors. The secondary aim of this study was to further investigate the impacts of the PMT-CBO intervention on children’s atopic diseases outcomes, including children’s medication adherence and subjective disease symptoms. We hypothesized that participants assigned to the PMT-CBO group would have significantly greater improvements in both the primary and secondary outcomes compared to those assigned to the control group, who only received routine verbal education during pediatric outpatient visits.

## Methods

### Study Design

This study, using a quasi-experimental design, was conducted in Hangzhou, China. Three health care institutions with high outpatient volumes were purposively selected from 3 districts of Hangzhou: a general hospital in Yuhang District (location Y), an integrated hospital in Xihu District (location X), and a general hospital in Gongshu District (location G). Locations Y and X were assigned to the PMT-CBO group, while location G served as the control group. This design intentionally substituted individual randomization with institutional-level assignment to reflect real-world implementation constraints and to control contamination risks through spatial segregation. Baseline equivalence testing confirmed comparable socioeconomic profiles across the groups.

### Recruiting Procedure

The sample size was calculated based on the primary outcome indicator, the protective motivation score of caregivers, using the formula for comparing the means of 2 independent groups:



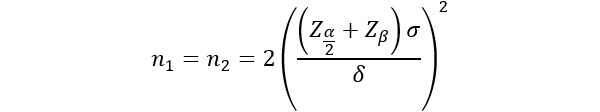



Where σ is the estimated population SD, *δ* represents the mean difference between groups, *Z_α/2_* is 1.96 for the significance level α of .05, and *Z_β_* is 1.28 for a test power of 80%. On the basis of prior cross-sectional survey data from the research group, the estimated overall protection motivation score was 3.50 (SD 0.47), yielding σ=0.47. Considering an anticipated increase of at least 0.30 points in the score (δ=0.30) after the PMT-CBO intervention, the estimated sample size was 52 for each group. Accounting for an estimated loss rate of 20%, the sample size was adjusted to a total of 126 individuals, with at least 63 in each group.

**Figure 2:**
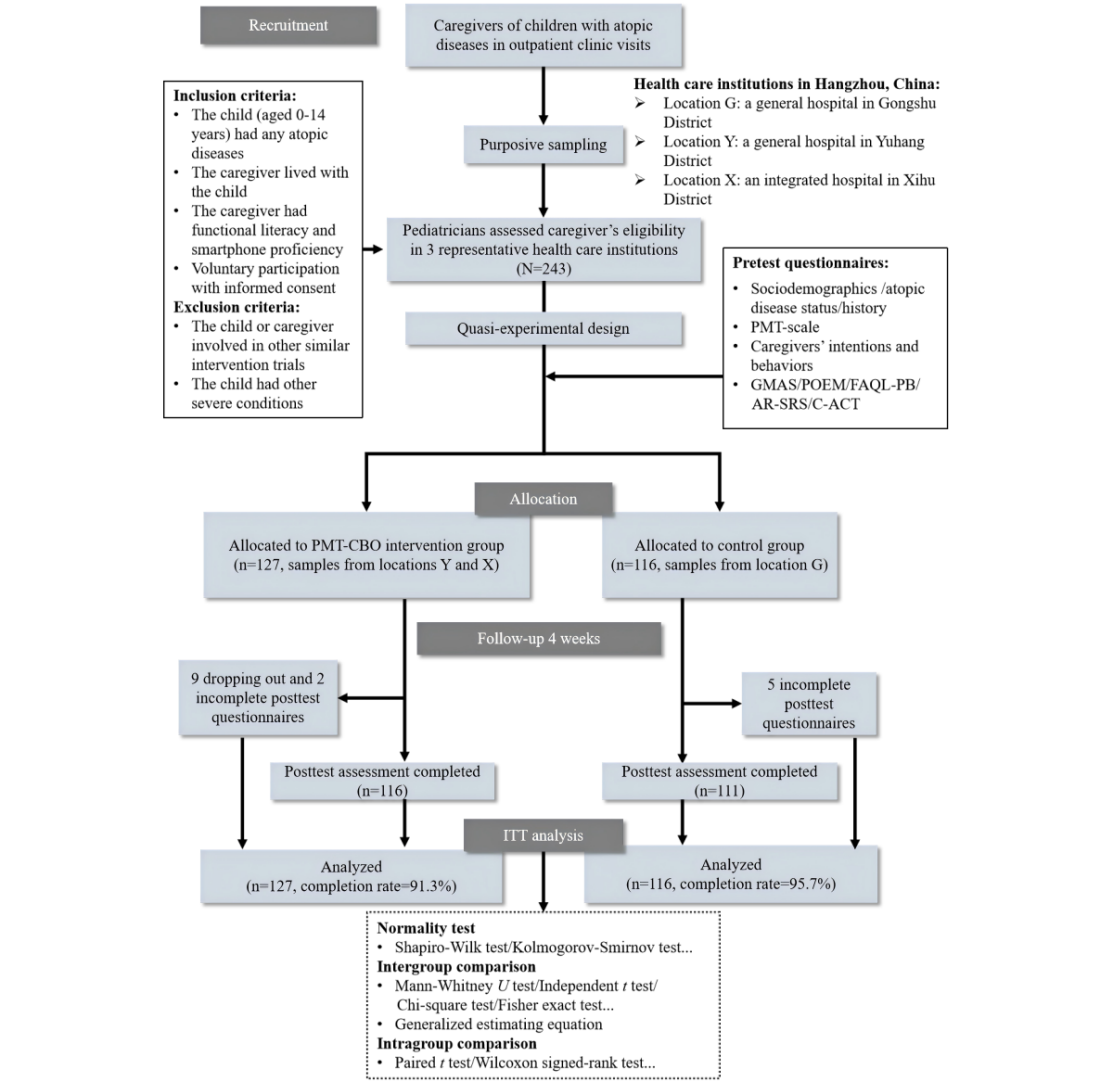
Flow diagram of participants through recruitment, assessment, allocation, follow-up, and intention-to-treat (ITT) analysis. AR-SRS: Allergic Rhinitis Symptom Rating Scale; C-ACT: Childhood Asthma Control Test; GMAS: General Medication Adherence Scale; PMT: Protection Motivation Theory; PMT-CBO: Protection Motivation Theory–based cognitive behavioral online; POEM: Patient-Oriented Eczema Measure.

Figure 2 illustrates the full study flow diagram. Recruitment was conducted between May and December 2023 during pediatric outpatient clinic visits for children with physician-diagnosed allergic diseases. Eligible families were approached by attending pediatricians to assess parental willingness to participate. Enrollment was voluntary, with caregivers providing written informed consent after receiving detailed study information. Caregivers were eligible if they (1) had at least one child diagnosed with atopic diseases, aged 0 to 14 years; (2) were the primary caregiver living with the child; (3) had functional literacy and smartphone proficiency; (4) and voluntarily participated and signed the consent form. Exclusion criteria included (1) participation in other cognitive behavioral intervention trials and (2) the child having severe physical or mental illnesses or complications requiring surgical treatment.

A total of 243 participants were enrolled and completed the baseline assessment. Of these, 127 (52.3%) from locations Y and X were allocated to the PMT-CBO group and 116 (47.7%) from location G were assigned to the control group.

### Ethical Considerations

The research tools, procedures, and consent forms were approved by the Ethics Review Committee of the School of Public Health, Hangzhou Normal University (approval no. 20230002). All participants signed the consent form and received a US $1.50 appreciation gift upon enrollment. All personal data in the study have been anonymized during the data organization and statistical analysis stages to ensure participant privacy.

### Intervention

#### PMT-CBO Intervention Group

The PMT-CBO intervention was developed by a multidisciplinary research team comprising 4 experts with >10 years of clinical experience in pediatric allergy, 3 health management specialists, and 5 graduate students who were majoring in health management or public health. The intervention consisted of 2 components: a WeChat chat group and a WeChat mini-program course. After enrollment, caregivers in the intervention group were invited to join a WeChat chat group by scanning a QR code with their mobile phones. Each chat group included 10 to 15 participants. Weekly learning tasks from the WeChat mini-program course were posted in the chat group to facilitate engagement.

The WeChat mini-program course had 16 modules of knowledge and skill training based on the principles of the PMT, aiming to educate caregivers of children with atopic diseases on effective condition management and attack prevention. Specifically, the 16 modules were organized into 4 chapters, each addressing specific dimensions of the PMT: (1) susceptibility and severity (chapter 1) to educate caregivers on the susceptibility and severity of childhood atopic diseases; (2) internal or external rewards (chapter 2) to address common misconceptions about prevention and treatment behaviors; (3) self-efficacy and response efficacy (chapter 3) to introduce practical methods and skills for daily prevention and treatment; and (4) response cost (chapter 4) to guide caregivers in overcoming challenges and barriers to managing atopic diseases. Specifically, Textbox 1 exhibits the list of educational content in accordance with the structure of the PMT, and Multimedia Appendix 1 provides the detailed timeline and protocol of the PMT-CBO intervention. Participants in the PMT-CBO intervention group were required to complete 1 chapter per week, over a 4-week intervention period.

To enhance the participation of caregivers and improve their adherence and completion rates during the intervention, the following incentive strategies were implemented. First, in the WeChat chat group, professional physicians provided individualized online consultations on child health to caregivers upon request, thereby encouraging their participation in the intervention. Second, participants’ progress in completing the mini-program course was monitored and announced weekly in the chat group. The research team sent reminders to caregivers who had not accessed the course content on time, thus encouraging task completion. Third, all participants were informed initially that they would be provided with various incentives (ie, saline nasal spray and antimite laundry detergent) at the end of the study. These incentives were based on their course completion status and activity level in the chat group, serving as a token of appreciation and encouragement.

Therefore, the 2 components of the PMT-CBO intervention, the WeChat chat group and mini-program course, functioned synergistically. Together, they provided caregivers with both general support and targeted guidance, creating a convenient and comprehensive framework to enhance caregiver knowledge and skills and improve disease management. In the PMT-CBO intervention group, the pre- and postintervention assessments were conducted at 2 time points: at enrollment and the fifth week after the intervention was completed.

**Educational content list based on the structure of the protection motivation theory.**

**Susceptibility**

- What are atopic diseases? What are the typical clinical manifestations of atopic diseases?
- What is the atopic march of atopic diseases?
- What is the prevalence of atopic diseases in China?
- Are atopic diseases hereditary? What kind of children are prone to atopic diseases?
- What environmental factors can trigger the onset of atopic diseases (hygiene hypothesis)?

**Severity**

- What behavioral factors can lead to the recurrence of atopic diseases?
- What factors can induce severe allergic reactions?
- What complications can arise from not actively treating atopic diseases?
- How can caregivers identify and urgently manage them?

**Self-efficacy**

- What are the common allergens? How to avoid them?
- What are tips for using antiallergic drugs according to medical prescription?
- How can children with atopic diseases arrange their diet reasonably?
- What sports can children with atopic conditions do? What are the precautions?
- How can families of children with atopic diseases live with the disease?
- Correct steps and precautions for nasal irrigation.
- Correct steps and precautions for nebulization.
- Correct steps and precautions for bathing and skin care.

**Response efficacy**

- How to scientifically understand allergen testing?
- Why does the treatment of atopic diseases need to be long-term?
- What are the short-term and long-term benefits of actively preventing and treating atopic diseases for children?
- What are the benefits of actively preventing and treating atopic diseases for families?
- How to distinguish atopic rhinitis and asthma from the common cold?
- How to distinguish eczema from common skin diseases such as chicken pox?
- How to choose the right hospital and department?
- What are the benefits of caregivers supervising their children’s medication and daily care?

**Response cost**

- Why does the child’s condition not improve after treatment?
- Why should an atopic diary be kept for children with atopic disease? How to assess the child’s condition?
- Can atopic diseases be cured?
- Should children with atopic diseases avoid certain foods?
- Can children with atopic diseases receive vaccinations? For example, COVID-19, influenza, and HPV vaccines.
- How to reduce unnecessary expenses related to atopic diseases?
- What is the safety of hormone treatment for atopic diseases, and why cannot hormones be stopped immediately after use?
- Why is allergen avoidance of great significance in the prevention and treatment of atopic diseases?

**Internal or external rewards**

- How to verify the scientific validity of atopic disease prevention and treatment information on the internet?
- Are probiotics promoted on the internet really effective in preventing and treating atopic diseases?
- Misconceptions in prevention and treatment behavior lecture—misconception one: basic cognitive misconceptions about atopic diseases.
- Misconceptions in prevention and treatment behavior lecture—misconception two: misconceptions about allergen avoidance and environmental control.
- Misconceptions in prevention and treatment behavior lecture—misconception three: misconceptions about the use of antiallergic drugs.
- Misconceptions in prevention and treatment behavior lecture—misconception four: misconceptions about medical treatment and referral.
- Misconceptions in prevention and treatment behavior lecture—misconception five: misconceptions about neglecting daily monitoring.
- Misconceptions in prevention and treatment behavior lecture—misconception six: misconceptions about daily care.

#### Control Group

Participants in the control group maintained their routine treatment and received verbal education during their pediatric outpatient visits following enrollment. The education content included basic knowledge about atopic diseases, specific explanations of the child’s condition, and personalized treatment plans. Pre- and postintervention assessments for the control group were conducted at 2 time points: at enrollment and at the fifth week after enrollment.

Following completion of the postintervention assessment, caregivers in the control group received a QR code via an SMS text message, enabling them to access the WeChat mini-program course used by the intervention group. This approach ensure the equity in resource availability across groups after the study.

### Measurements

#### Sociodemographic and Atopic Disease-Related Information Measures

The sociodemographic and atopic disease-related information questionnaire was designed by the research team and included two parts: (1) the child’s personal information and conditions, such as age, gender, type of atopic disease, height, weight, feeding type within 1 year of age, and severity and frequency of attacks of disease in the past year; (2) caregiver’s sociodemographic information, such as caregiver identity, age, household registration, highest level of education, occupation, family monthly income, history of atopic diseases, and birth order of the child.

#### Primary Outcomes of Caregiver’s Protection Motivation, Intentions, and Behaviors

##### Atopic Disease Protection Motivation Scale for Caregivers of Children With Atopic Disease

To measure the protective motivation of caregivers of children with atopic diseases, the PMT scale was developed by the research team in a previous cross-sectional study using data from a pilot survey (N=440) and validated in an empirical study (N=443). The scale demonstrated good reliability (Cronbach α=0.84) and validity (χ*^2^/df*=2.918, root mean square error of approximation=0.066, goodness of fit index=0.906, comparative fit index=0.937, normed fit index=0.908, incremental fit index=0.937, Tucker-Lewis index=0.919) in the empirical stage. The scale consists of 6 dimensions: caregivers’ perceived susceptibility and severity of allergic diseases, self-efficacy, response efficacy, response cost, and internal or external rewards related to the prevention and management of children’s atopic conditions. The items of the scale are described in Multimedia Appendix 2. Participants rated their agreement with each item on a 5-point Likert scale, ranging from “strongly disagree” (1 point) to “strongly agree” (5 points).

##### Caregiver’s Intentions and Behaviors Regarding Prevention and Control of Children’s Atopic Diseases

The general and specific questions of intentions and behaviors related to caregivers’ prevention and control of children’s atopic diseases were self-developed and assessed using a 5-point questionnaire, where higher ratings indicated greater intentions and more frequent behaviors. For intentions, the scale ranged from “very willing” (5 points) to “very unwilling” (1 point). For behaviors, the scale ranged from “always” (5 points) to “never” (1 point). Detailed descriptions of the questionnaire are presented in Multimedia Appendix 2.

#### Secondary Outcomes of Children’s Subjective Disease Symptoms and Medication Adherence

##### Food Allergy Quality of Life–Parental Burden Scale

The Food Allergy Quality of Life–Parental Burden (FAQL-PB) scale for patients with food allergies was originally developed by Cohen et al [51]. The cross-cultural adaptation for the Chinese version strictly followed internationally accepted guidelines [52]. The scale consists of 17 items, addressing the impact of food allergies on various family and social dimensions. Items are rated on a 7-point Likert scale (0-6), reflecting the caregiver’s level of worry or distress. The total score ranges from 0 to 102, with higher scores indicating greater parental burden and poorer quality of life. The Chinese version of FAQL-PB has demonstrated strong reliability and validity, with a Cronbach α coefficient of 0.97, a Kaiser-Meyer-Olkin measure of 0.94, and Bartlett χ^2^_136_=4366.8 (*P<*.05) [52].

##### Allergic Rhinitis Symptom Rating Scale

The Allergic Rhinitis Symptom Rating Scale (AR-SRS) is based on the Expert Consensus on Mite-Specific Immunotherapy for Childhood Airway Allergic Diseases and the Expert Consensus on the Diagnosis, Treatment, and Prevention of Allergic Diseases (III) [53,54]. This scale assesses the severity of rhinitis symptoms, including 4 key nasal symptoms: sneezing, runny nose, nasal congestion, and nasal itching. The total score ranges from 0 to 12, with higher scores indicating more severe symptoms.

##### Patient-Oriented Eczema Measure

The Patient-Oriented Eczema Measure (POEM) for patients with eczema was originally developed by Charman et al [55] and subsequently translated into Chinese by the atopic dermatitis team of the Guangdong Provincial Hospital of Traditional Chinese Medicine. The Chinese version of the scale, designed for caregivers to complete on behalf of children, has been validated for good reliability and validity, with a Cronbach α coefficient of 0.81, Kaiser-Meyer-Olkin measure of sampling adequacy of 0.79, and Bartlett test of sphericity yielded a *χ^²^*_21_ value of 250.69 (*P*<.001) [56]. The total score ranges from 0 to 28, with higher scores indicating more severe eczema symptoms.

##### Childhood Asthma Control Test

The Childhood Asthma Control Test (C-ACT) is a questionnaire recommended by the Global Initiative for Asthma to assess asthma control in children aged 4 to 11 years [57]. The Chinese version of C-ACT consists of 7 items with a total score ranging from 0 to 27. Higher scores indicate better asthma control. It has been validated and shown to have good reliability and validity, with a Cronbach α coefficient of 0.76 and significant correlation with physician evaluations of asthma control (*r*²=0.61; *P*<.001) [58].

##### General Medication Adherence Scale

The Chinese version of the General Medication Adherence Scale (GMAS) consists of 11 items across 3 dimensions. The original version was developed by Naqvi et al [59] in 2018, and the Chinese translation was completed by Wang et al [60] in 2021. The 3 dimensions included patient behavior-related adherence (5 items), additional disease and medication burden (4 items), and cost-related adherence (2 items). The scale has been validated, with a Cronbach α coefficient of 0.78 and a test-retest reliability coefficient of 0.88 and an item-level content validity index of ≥0.78 indicating good reliability and validity [60]. The score ranges from 0 to 33, with higher scores reflecting better medication adherence.

### Statistical Analysis

The normality tests were conducted on continuous variables using the Shapiro-Wilk test for samples with n≤50 or the Kolmogorov-Smirnov test for samples with n>50. The normally distributed continuous variables were presented as mean (SD), nonnormally distributed continuous variables were expressed as median (IQR), while categorical variables and ordinal variables were reported as counts (percentages). Intergroup differences were evaluated using the independent *t* tests for normally distributed continuous variables, Mann-Whitney *U* tests for nonnormally distributed continuous or ordinal variables, and chi-square or Fisher exact test for categorical variables. Intragroup differences between pre- and postintervention measures were examined with paired 2-tailed *t* tests for normally distributed variables and Wilcoxon signed-rank tests for nonnormally distributed continuous variables. The statistical significance level for all tests was set at *P*<.05 (2-tailed).

Given that the evaluation metrics of protection motivation were nonnormally distributed, and to control for potential confounders that might influence the evaluation of the intervention’s effect, the study used a multivariable generalized estimating equation (GEE) model with an exchangeable correlation structure adjusting for impactful demographic characteristics. In this model, the various dimensions of protective motivation and the overall PMT score were treated as individual outcome variables. The group assignment (experimental vs control) and time point (after the intervention vs before the intervention) served as the exposure variables. To capture how the potential intervention effect might change with time, an interaction term between time and group was also incorporated. Furthermore, demographic and disease-related factors were included as potential confounders, including the child’s age, sex, caregiver identity, household registration status, parental history of atopic diseases, and the child’s birth order within the family. These confounders (eg, child’s age, sex, parental history of atopic diseases, child’s birth order within the family [61,62], and the caregiver identity [6,63]) were selected based on prior literature documenting risk factors for pediatric atopic disease prevention and control. Household registration status was used as a proxy for environmental determinants and health care accessibility [4,64]. On the basis of the data distribution characteristics, the Gamma GEE with a log link function and type III analysis was used to analyze nonnormally distributed continuous variables after the 4-week intervention. Statistical analyses were performed with SPSS (version 27.0; IBM Corp) and R (version 4.3.1; R Foundation for Statistical Computing).

## Results

### Baseline Characteristics

After a 4-week intervention, a total of 116 participants from the experimental group and 111 participants from the control group completed the posttest questionnaire, with a completion rate of 91.3% (116/127) and 95.7% (111/116), respectively (Figure 2). A chi-square test was conducted to investigate whether there was a significant difference between the control group and the experimental group in terms of lost visits during the intervention process (*χ*^2^_1_=0.1; *P*=.80). These results showed that the number of participants was still balanced between the 2 groups after the intervention, with no significant difference in their rates of lost visits. All data analysis were performed using intention-to-treat analysis to reflect the true effects of the intervention under the quasi-experimental design and to minimize the influence of selection bias on the study conclusions (Table 1).

**Table 1:** The number of study participants in the control and protection motivation theory–based cognitive behavioral online intervention (PMT-CBO) groups during the intervention phase.

Group	Baseline	After the intervention	Chi-square (*df*)	*P* value
Control	116	111	0.1 (1)	.80
PMT-CBO	127	116	0.1 (1)	.80

The baseline characteristics of the children recruited in the study and their subjective disease symptoms and medication adherence status are summarized in Tables 2 and 3. The median age of the allergic children was 4.00 (IQR 3.00-7.00) years, average height was 109.79 (SD 20.52) cm, and median weight was 18.50 (IQR 15.00-25.00) kg, with 63% (153/243) of them being boys. Among the types of atopic diseases, most children (103/243, 42.4%) were affected by allergic rhinitis, while a substantial proportion (103/243, 42.4%) were also reported to have more than 1 atopic disease. Regarding self-reported disease symptoms and medication adherence status, the median or mean scores for POEM, FAQL-PB, AR-SRS, and C-ACT were 7.00 (IQR 5.00-9.00), 2.00 (IQR 1.88-2.35), 4.00 (IQR 3.00-5.00), and 19.46 (SD 3.48), respectively, and the median score for GMAS was 25.00 (IQR 24.00-26.00), indicating relatively poor symptom control and a low quality of life for caregivers.

**Table 2:** Baseline characteristics of children.

Characteristics			Overall (N=243)		Control (n=116)		PMT-CBO^a^ (n=127)		*P* value	
Age (y), median (IQR)			4.00 (3.00-7.00)		5.00 (3.00-7.00)		4.00 (3.00-7.00)		.31^b^	
Height (cm), mean (SD)			109.79 (20.52)		111.72 (20.70)		108.02 (20.27)		.16^c^	
Weight (kg), median (IQR)			18.50 (15.00-25.00)		19.75 (15.00-25.00)		18.00 (14.00-24.00)		.28^b^	
**Sex, n (%)**										.99^d^
	Male	153 (63)		73 (62.9)		80 (63)				
	Female	90 (37)		43 (37.1)		47 (37)				
**Feeding type within 1 year of age, n (%)**										.57^d^
	Breastfeeding	125 (51.4)		57 (49.1)		68 (53.5)				
	Formula feeding	48 (19.8)		26 (22.4)		22 (17.3)				
	Mixed feeding	69 (28.4)		32 (27.6)		37 (29.1)				
	Other	1 (0.4)		1 (0.9)		0 (0)				
**The type of atopic disease, n (%)**										.06^d^
	Atopic dermatitis	7 (2.9)		3 (2.6)		4 (3.1)				
	Food allergic	14 (5.8)		10 (8.6)		4 (3.1)				
	Allergic rhinitis	103 (42.4)		55 (47.4)		48 (37.8)				
	Asthma	16 (6.6)		4 (3.4)		12 (9.4)				
	Comorbidity, ≥2 types of atopic diseases	103 (42.4)		44 (37.9)		59 (46.5)				
**Disease severity in the past year, n (%)**										.63^b^
	Very severe	2 (0.8)		0 (0)		2 (1.6)				
	Severe	84 (34.6)		44 (37.9)		40 (31.5)				
	Moderate	104 (42.8)		46 (39.7)		58 (45.7)				
	Mild	46 (18.9)		25 (21.6)		21 (16.5)				
	Very mild	7 (2.9)		1 (0.9)		6 (4.7)				
**The frequency of attacks in the past year, n (%)**										.42^b^
	Almost no attacks	12 (4.9)		7 (6.0)		5 (3.9)				
	1-2 attacks in half a year	72 (29.6)		37 (31.9)		35 (27.6)				
	3-4 attacks in half a year	79 (32.5)		34 (29.3)		45 (35.4)				
	1 attack per month	62 (25.5)		31 (26.7)		31 (24.4)				
	≥2 attacks per month	18 (7.4)		7 (6.0)		11 (8.7)				

^a^PMT-CBO: protection motivation theory–based cognitive behavioral online intervention.

^b^Mann-Whitney *U* test.

^c^Independent samples *t* test.

^d^Chi-square test or Fisher exact test.

**Table 3:** Comparison of preintervention subjective symptom and medication adherence scores in children with atopic diseases.

Atopic disease symptom and medication adherence score scales and group		Participants, n	Scores, median (IQR) or mean (SD)	*P* value	
**POEM^a^**					.77
	Overall	39	7.00 (5.00-9.00)^b^		
	PMT-CBO^c^	21	7.00 (4.00-12.00)^b^		
	Control	18	6.50 (5.00-9.00)^b^		
**FAQL-PB^d^**					.06
	Overall	59	2.00 (1.88-2.35)^b^		
	PMT-CBO	25	2.29 (1.94-2.41)^b^		
	Control	34	1.94 (1.81-2.29)^b^		
**AR-SRS^e^**					.25
	Overall	195	4.00 (3.00-5.00)^b^		
	PMT-CBO	103	4.00 (3.00-5.00)^b^		
	Control	92	4.00 (3.00-4.00)^b^		
**C-ACT^f^**					.80^g^
	Overall	69	19.46 (3.48)^h^		
	PMT-CBO	47	19.38 (3.79)^h^		
	Control	22	19.62 (2.75)^h^		
**GMAS^i^**					.09
	Overall	243	25.00 (24.00-26.00)^b^		
	PMT-CBO	127	25.00 (24.00-27.00)^b^		
	Control	116	25.00 (24.00-26.00)^b^		

^a^POEM: Patient-Oriented Eczema Measure.

^b^Median (IQR).

^c^PMT-CBO: protection motivation theory–based cognitive behavioral online intervention.

^d^FAQL-PB: Food Allergy Quality of Life–Parental Burden.

^e^AR-SRS: Allergic Rhinitis Symptom Rating Scale.

^f^C-ACT: Childhood Asthma Control Test.

^g^Independent samples *t* test, otherwise Mann-Whitney *U* test.

^h^Mean (SD).

^i^GMAS: General Medication Adherence Scale.

The baseline characteristics of the caregivers are presented in Table 4. Among the caregivers, most of the participants were mothers, comprising 76.5% (186/243) of the sample. At baseline, the caregivers’ protective motivation regarding their children’s atopic diseases was assessed using a developed and validated PMT scale. This scale measured 6 separate dimensions of protection motivation, with each dimension scored on a scale ranging from 0 to 100. The median scores for the 6 dimensions were as follows: susceptibility, 83.33 (IQR 75.00-91.67); severity, 75.00 (IQR 75.00-83.33); self-efficacy, 58.33 (IQR 50.00-66.67); response efficacy, 78.57 (IQR 75.00-85.71); response cost, 41.67 (IQR 33.33-50.00); and internal or external rewards, 41.67 (IQR 33.33-50.00). The overall PMT score achieved a median score of 65.91 (IQR 61.36-69.32), far from the maximum score of 100. At baseline, no substantial differences were observed between the intervention and control groups concerning caregivers’ demographics, protective motivation, or children’s demographics, severity and types of atopic diseases, frequency of attacks, subjective symptoms, and medication adherence.

**Table 4:** Baseline characteristics of caregivers in the study.

Characteristics		Overall (N=243)	Control (n=116)	PMT-CBO^a^ (n=127)	*P* value
Age (y), median (IQR)		35.00 (32.00-39.00)	35.00 (33.00-38.00)	35.00 (31.00-39.00)	.21^b^
**The identity of the caregiver, n (%)**					.20^c^
	Father	44 (18.1)	26 (22.4)	18 (14.2)	
	Mother	186 (76.5)	83 (71.6)	103 (81.1)	
	Grandparents	13 (5.3)	7 (6.0)	6 (4.7)	
**Household registration, n (%)**					.46^c^
	Urban	179 (73.7)	88 (75.9)	91 (71.7)	
	Rural	64 (26.3)	28 (24.1)	36 (28.3)	
**Highest level of education, n (%)**					.85^b^
	Primary school and below	10 (4.1)	7 (6.0)	3 (2.4)	
	Junior high school	14 (5.8)	4 (3.4)	10 (7.9)	
	High school or technical secondary	30 (12.3)	14 (12.1)	16 (12.6)	
	Technical or vocational school	61 (25.1)	30 (25.9)	31 (24.4)	
	Bachelor’s degree	112 (46.1)	52 (44.8)	60 (47.2)	
	Master’s degree or higher	16 (6.6)	9 (7.8)	7 (5.5)	
**Occupation, n (%)**					.99^c^
	Public sector employee	33 (13.6)	16 (13.8)	17 (13.4)	
	Private sector employee	119 (49.0)	58 (50.0)	61 (48.0)	
	Self-employed and freelancers	28 (11.5)	13 (11.2)	15 (11.8)	
	Agricultural workers	6 (2.5)	3 (2.6)	3 (2.4)	
	Homemakers	46 (18.9)	21 (18.1)	25 (19.7)	
	Other	11 (4.5)	5 (4.3)	6 (4.7)	
**Family monthly income (RMB^d^, n (%)**					.32^b^
	≤5000	4 (1.6)	0 (0)	4 (3.1)	
	5001-10,000	47 (19.3)	23 (19.8)	24 (18.9)	
	10,001-15,000	132 (54.3)	71 (61.2)	61 (48.0)	
	15,001-20,000	33 (13.6)	13 (11.2)	20 (15.7)	
	>20,000	27 (11.1)	9 (7.8)	18 (14.2)	
**History of atopic disease, n (%)**					.15^c^
	Neither parent has atopic disease	90 (37)	50 (43.1)	40 (31.5)	
	One parent has atopic disease	110 (45.3)	49 (42.2)	61 (48)	
	Both parents have atopic disease	43 (17.7)	17 (14.7)	26 (20.5)	
**Birth order of the child with atopic disease, n (%)**					.13^b^
	First	173 (71.2)	88 (75.9)	85 (66.9)	
	Second	63 (25.9)	25 (21.6)	38 (29.9)	
	Third	7 (2.9)	3 (2.6)	4 (3.1)	
**Protection motivation score, median (IQR)**					
	Susceptibility	83.33 (75.00-91.67)	75.00 (75.00-83.33)	83.00 (66.67-91.67)	.10^b^
	Severity	75.00 (75.00-83.33)	75.00 (75.00-83.33)	75.00 (75.00-83.33)	.67^b^
	Self-efficacy	58.33 (50.00-66.67)	58.33 (50.00-66.67)	58.33 (50.00-66.67)	.38^b^
	Response efficacy	78.57 (75.00-85.71)	82.14 (75.00-85.71)	78.57 (75.00-89.29)	.93^b^
	Response cost	41.67 (33.33-50.00)	41.67 (33.33-41.67)	41.67 (33.33-50.00)	.76^b^
	Internal or external rewards	41.67 (33.33-50.00)	41.67 (33.33-50.00)	50.00 (25.00-58.33)	.12^b^
	Overall PMT^e^ score	65.91 (61.36-69.32)	65.91 (62.50-68.18)	65.91 (60.23-72.73)	.30^b^

^a^PMT-CBO: protection motivation theory–based cognitive behavioral online intervention.

^b^Mann-Whitney *U* test.

^c^Chi-square test.

^d^1 RMB=US$ 0.14.

^e^PMT: protection motivation theory.

### Changes in Caregiver’s Protection Motivation During the Follow-Up Period

**Figure 3:**
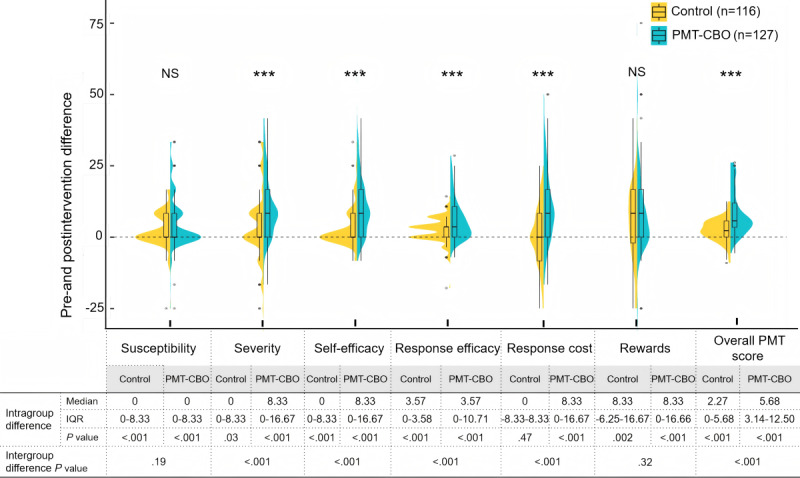
Comparison of protective motivation scores of caregivers in the Protection Motivation Theory–based cognitive behavioral online (PMT-CBO) group and the control group before and after the intervention. Rewards refer to internal or external rewards. NS: no significance; PMT-CBO: Protection Motivation Theory–based cognitive behavioral online intervention. ***P<.001.

First, both changes of PMT scores within the PMT-CBO group before and after the intervention and the changes within the control group were calculated and are presented in Figure 3 and Multimedia Appendix 3, which are illustrated as intragroup differences. It demonstrated that, following the intervention, the scores across all 6 dimensions of protective motivation, as well as the overall PMT score, significantly increased in the PMT-CBO group compared to the preintervention (*P*<.001). Among them, the scores for the dimensions of severity (median 8.33, IQR 0-16.67), self-efficacy (median 8.33, IQR 0-16.67), response cost (median 8.33, IQR 0-16.67), and internal or external rewards (median 8.33, IQR 0-16.66) increased substantially (see Multimedia Appendix 3 for details). This finding indicates a substantial enhancement in the protective motivation of caregivers in the PMT-CBO group after the intervention. Moreover, in the control group, postintervention scores were significantly higher than preintervention scores across most PMT dimensions except for the response cost dimension (*P*<.05; Figure 3 and Multimedia Appendix 3). Second, a comparison analysis of the score difference between the 2 groups before and after the intervention, illustrated as intergroup difference in Figure 3, revealed that the PMT-CBO group exhibited significantly greater improvements than the control group in both the overall PMT score (median 5.68, IQR 3.41-12.50 vs median 2.27, IQR 0-5.68) and most of individual dimensions (*P*<.001), excluding the dimensions of susceptibility and internal or external rewards.

The results of the multivariable GEE models to assess changes in the dimensions of protective motivation and the overall score from before to after the intervention are presented in Table 5. It indicated that, according to the effect of the group-time interaction term (*β*=.027; *P*=.03), the PMT-CBO intervention based on the PMT implemented in the experimental group showed a significant positive impact on the susceptibility dimension compared to the routine intervention received by the control group. Similarly, the experimental group demonstrated significantly greater improvements in the dimensions of severity, self-efficacy, response efficacy, and response cost, as well as in the overall protective motivation score (*P*<.001), with response cost obtaining the highest effect of group-time interaction (*β*=.231). These findings suggested that, even after controlling for potential confounding factors, the cognitive behavioral online intervention effectively enhanced caregivers’ knowledge of and proactive engagement in managing pediatric atopic diseases. However, in the specific dimension of internal or external rewards, no significant interaction effect was observed between time and group (*β*=.005; *P*=.90), indicating that the improvement in this dimension in the experimental group was not substantially different from that in the control group.

**Table 5:** Generalized estimating equation model effect estimation results.

Dimension	Groups effects^a^			Time^b^			Interaction of group×time^c^	
	β (SE)	*P* value^d^	β (SE)		*P* value	β (SE)		*P* value
Susceptibility	.016 (0.024)	.50	.034 (0.007)		<.001	.027 (0.013)		.03
Severity	.007 (0.021)	.73	.036 (0.017)		.03	.098 (0.021)		<.001
Self-efficacy	.030 (0.030)	.32	.049 (0.010)		<.001	.118 (0.018)		<.001
Response efficacy	.016 (0.014)	.26	.030 (0.005)		<.001	.042 (0.009)		<.001
Response cost	.016 (0.033)	.63	.014 (0.024)		.56	.231 (0.034)		<.001
Internal or external rewards	.129 (0.041)	.002	.103 (0.030)		<.001	.005 (0.041)		.90
Overall protection motivation theory score	.018 (0.012)	.14	.038 (0.005)		<.001	.079 (0.011)		<.001

^a^The groups are the experimental group and the control group (reference group).

^b^The time refers to postintervention and preintervention (reference group).

^c^The reference group between groups×time is the preintervention×control group.

^d^The generalized estimating equation models were adjusted for the child’s age and sex, caregiver’s identity, household registration, caregiver’s history of atopic disease, and birth order of the child with atopic diseases.

### Change of Caregivers’ Intentions and Behavior Frequency of Prevention and Control During the Follow-Up Period

In this study, we also assessed both intra- and intergroup changes in caregivers’ intentions to prevent and control their child’s atopic conditions. As shown in Table 6, after 4 weeks of intervention, caregivers in the PMT-CBO group exhibited significantly higher levels of proactivity and improvement compared to the control group across most key indicators of prevention and control intentions (*P*<.05). Specifically, significant improvements were observed in the intentions to prevent their child’s allergic diseases in general (*Z*=–2.665; *P*=.008); use corticosteroid-based antiallergic medications (*Z*=–3.442; *P*<.001); control allergens regularly (*Z*=–3.025; *P*=.002); receive health education related to allergic diseases (*Z*=–2.403; *P*=.02); seek medical treatment promptly and adhere to prescribed medications (*Z*=–2.275; *P*=.02); and attend follow-up appointments regularly (*Z*=–2.202; *P*=.03). However, no significant improvement was observed in caregivers’ intentions to monitor their child’s condition regularly when compared to the control group (*Z*=–1.873; *P*=.06).

**Table 6:** Comparison of caregivers’ intentions for prevention and treatment between the 2 groups before and after the intervention.

Intention-related items and groups			Intragroup					Intergroup										
			*Z* value		*P* value^a^		Preintervention							Postintervention				
							*Z* value				*P* value^b^			*Z* value			*P* value^b^	
**Intention to prevent your child’s allergic diseases in general**									–1.913			.06			–2.665			.008
	PMT-CBO^c^	–1.720		.09														
	Control	–0.955		.34														
**Intention to seek medical treatment promptly and attend follow-up appointments regularly**									–1.898			.06			–2.202			.03
	PMT-CBO	–2.696		.007														
	Control	–0.757		.45														
**Intention to adhere to appropriate medication as prescribed by your child’s doctor**									–0.876			.38			–2.275			.02
	PMT-CBO	–4.327		<.001														
	Control	–0.890		.37														
**Intention to use corticosteroid-based antiallergic medications for your child**									–1.120			.26			–3.442			<.001
	PMT-CBO	–7.026		<.001														
	Control	–3.376		.001														
**Intention to undergo regular follow-up visits for your child’s allergic diseases**									–0.424			.67			–2.040			.04
	PMT-CBO	–3.175		.001														
	Control	–0.453		.65														
**Intention to control allergens regularly**									–1.030			.30			–3.025			.002
	PMT-CBO	–3.523		<.001														
	Control	–1.053		.29														
**Intention to monitor your child’s condition regularly**									–0.474			.64			–1.873			.06
	PMT-CBO	–3.402		<.001														
	Control	–0.451		.65														
**Intention to receive health education related to allergic diseases**									–1.185			.24			–2.403			.02
	PMT-CBO	–3.667		<.001														
	Control	–0.443		.66														
**Intention to participate in health education online regularly**									–0.078			.94			–3.103			.002
	PMT-CBO	–3.868		<.001														
	Control	–0.746		.46														

^a^Wilcoxon signed-rank test.

^b^Mann-Whitney *U* test.

^c^PMT-CBO: protection motivation theory–based cognitive behavioral online intervention.

In terms of behavioral outcomes related to the prevention and control of atopic diseases, caregivers in the PMT-CBO group reported lower incidences of their child’s atopic diseases due to allergen exposure compared to those in the control group both before (*Z*=–2.429; *P*=.02) and after the 4-week intervention (*Z*=–2.396; *P*=.02; Multimedia Appendix 4). In addition, a significantly higher percentage of caregivers in the PMT-CBO group attended health education sessions compared to the control group (*Z*=–2.149; *P*=.03), likely as a direct result of the PMT-CBO intervention. Within the PMT-CBO group, significant improvements were observed after the intervention in behaviors such as attending follow-up appointments regularly (*Z*=–2.371; *P*=.02) and adhering to prescribed medication regimens (*Z*=–2.077; *P*=.04). However, no significant inter- or intragroup differences were observed for other prevention and control behaviors, such as adopting general preventive measures for atopic diseases (*Z*=–1.016; *P*=.31) or monitoring the child’s condition regularly (*Z*=–0.888; *P*=.37). These findings may be attributed to the relatively short duration of the intervention, which might have been insufficient to elicit substantial behavioral change.

### Changes in Medication Adherence of Children During the Follow-Up Period

**Figure 4:**
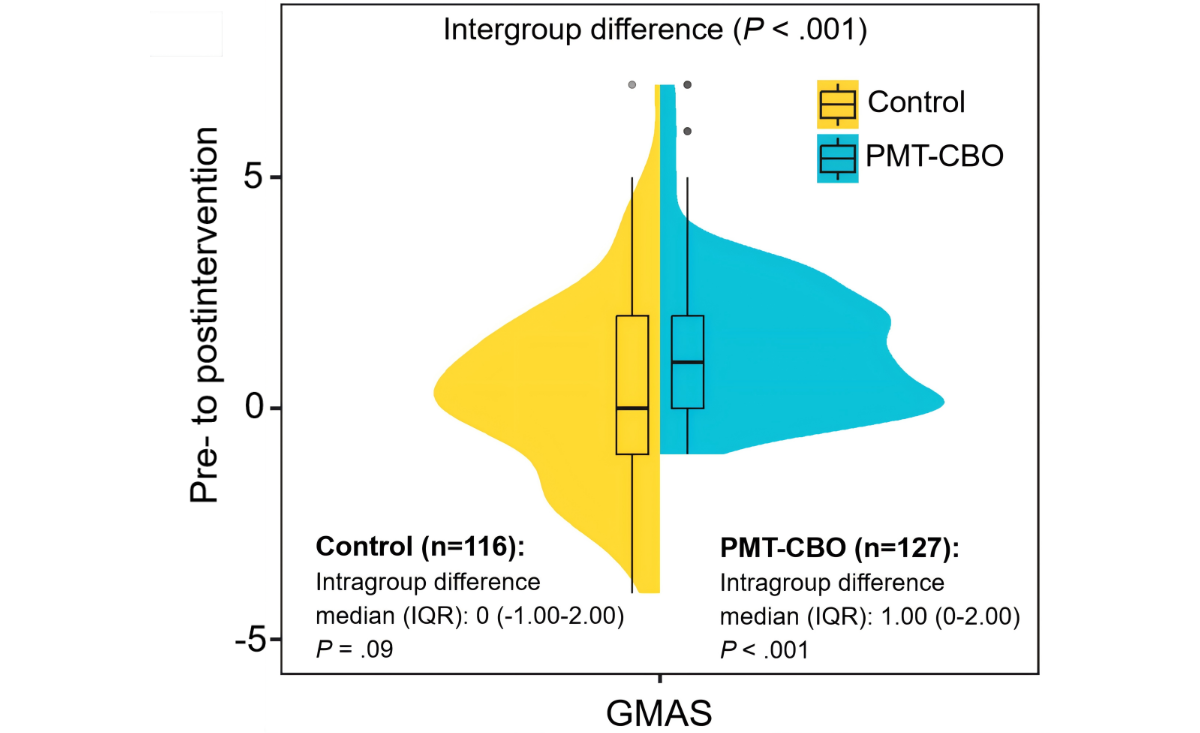
Comparison of medication adherence of children with atopic diseases in the Protection Motivation Theory–based cognitive behavioral online (PMT-CBO) group and the control group before and after the intervention. A Mann-Whitney U test determined *P*<.001 in the intergroup difference. The Wilcoxon signed-rank test was conducted for the PMT-CBO group and determined the *P* values in the control and PMT-CBO groups. GMAS: General Medication Adherence Scale.

Within the PMT-CBO group, medication adherence as measured by GMAS improved significantly after the intervention compared to the preintervention period (*Z*=–7.970; *P*<.001; Figure 4 and Multimedia Appendix 5). The median difference in GMAS scores between before and after the intervention for the PMT-CBO group was 1.00 (IQR 0-2.00). In contrast, no significant improvement in medication adherence was observed in the control group following routine care and verbal education (*Z*=–1.673; *P*=.09; see Multimedia Appendix 5 for details). Furthermore, when comparing the 2 groups, the improvement in children’s medication adherence scores in the PMT-CBO group was significantly greater than that in the control group (*Z*=–4.457; *P*<.001). These findings highlight the significant effect of the PMT-CBO intervention in improving children’s medication adherence through caregiver engagement.

### Changes in Subjective Symptoms of Atopic Diseases in Children During the Follow-Up Period

**Figure 5:**
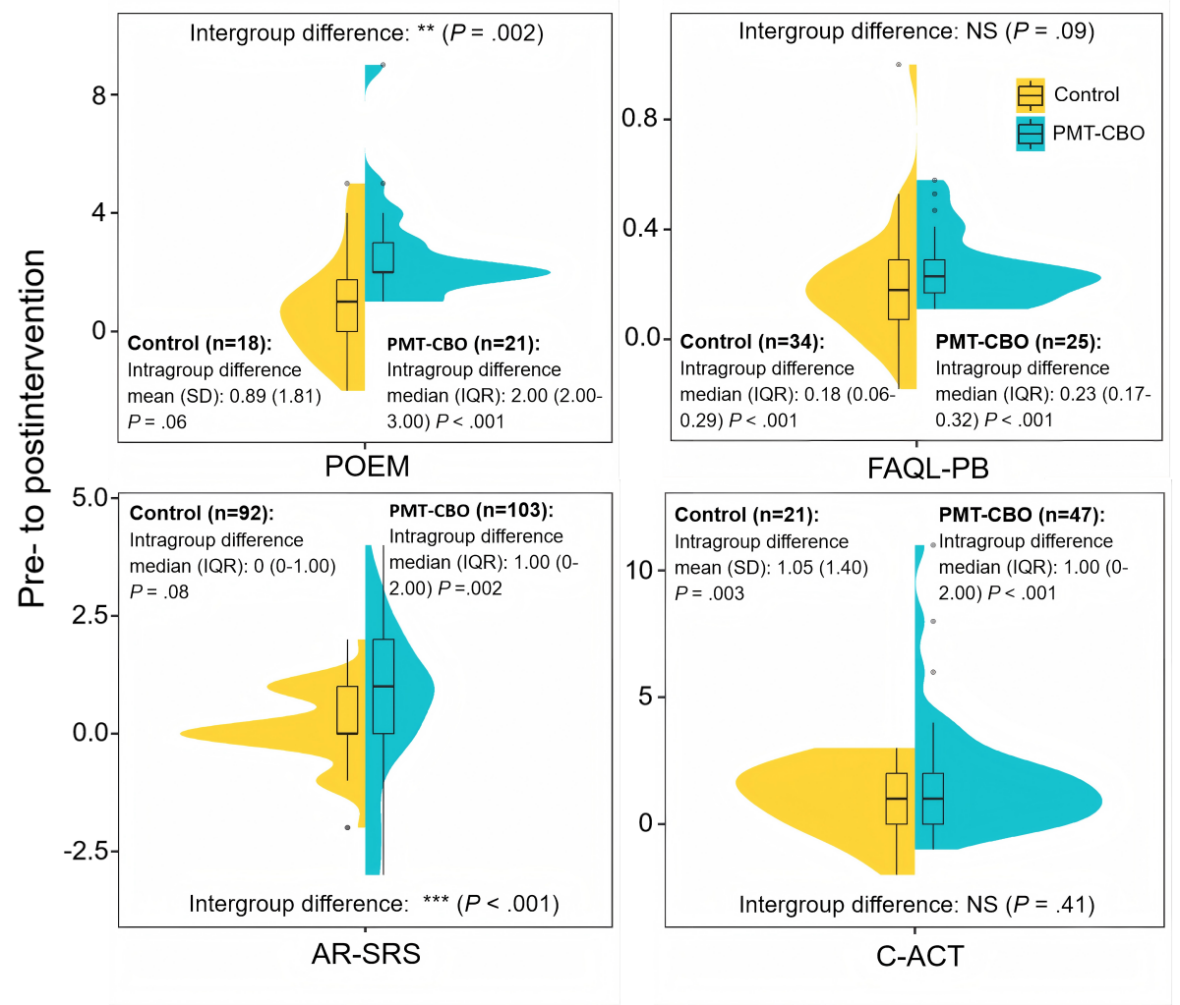
Comparison of subjective atopic disease symptom scores of children with atopic diseases in the 2 groups before and after the intervention. AR-SRS: Allergic Rhinitis Symptom Rating Scale; C-ACT: Childhood Asthma Control Test; FAQL-PB: Food Allergy Quality of Life–Parental Burden; NS: no significance; PMT-CBO: the Protection Motivation Theory–based cognitive behavioral online intervention; POEM: Patient-Oriented Eczema Measure. **P<.01, ***P<.001.

The results presented in Figure 5 indicate that, compared to the preintervention period, children in the PMT-CBO group showed a significant decline in POEM, FAQL-PB, and AR-SRS scores following the intervention (all *P* values <.01). For instance, the median difference in POEM scores between before and after the intervention for the PMT-CBO group was 2.00 (IQR 2.00-3.00). These reflected improvements in children’s eczema symptoms, caregiving burden for caregivers of children with food allergies, and children’s allergic rhinitis symptoms. Meanwhile, the C-ACT score significantly increased in the PMT-CBO group (*t*_46_=–5.582; *P*<.001), with a median score difference of 1.00 (IQR 0-2.00), indicating a marked improvement in asthma control in these children. In the control group, significant improvements were observed only in FAQL-PB (*Z*=–4.307; *P*<.001) and C-ACT (*t*_20_=–3.440; *P*=.003) scores (see Multimedia Appendix 6 for details).

More importantly, in terms of intergroup differences, the PMT-CBO group exhibited significantly greater improvements in POEM (*Z*=–3.112; *P*=.002) and AR-SRS (*Z*=–3.277; *P*<.001) scores compared to the control group (see Figure 5 and Multimedia Appendix 6 for details). However, no significant intergroup differences were observed for changes in FAQL-PB (*Z*=–1.693, *P*=.09) and C-ACT scores (*Z*=–.830, *P*=.41). These findings suggested that the PMT-CBO intervention effectively improved eczema and allergic rhinitis symptoms in children.

## Discussion

### Principal Findings

This study demonstrated that the 4-week PMT-CBO intervention yielded significant intragroup improvements in caregivers’ overall PMT scores and all PMT dimensions in pre-post comparisons. Intergroup comparisons confirmed the superior efficacy of PMT-CBO group over controls in most PMT dimensions, except for susceptibility and internal or external reward dimensions. Furthermore, caregivers receiving the PMT-CBO intervention displayed better outcomes in key indicators of atopic prevention and control intentions, alongside partial improvements in relevant behavioral measures related to disease management. Regarding child-level outcomes, children in the PMT-CBO group demonstrated improved intergroup medication adherence and clinically meaningful alleviation of eczema and allergic rhinitis symptoms compared to the control group, while no significant differences emerged in asthma control metrics or caregiver life burden associated with food allergy management.

### PMT Theoretical Mechanisms and Intrinsic Linkages

Among 6 PMT dimensions, self-efficacy and response efficacy exhibited the most pronounced intergroup enhancements in our study, which was aligned with previous PMT-based studies of caregivers of children with asthma [34]. The marked elevation in self-efficacy and response efficacy likely stemmed from the intervention’s skill-building components, such as structured guidance on allergen avoidance, medication adherence, and daily care routine and educational content emphasizing the benefits of adherence. This was consistent with findings from the trial by Lima et al [20], where education content contains topics such as the following: “Let’s learn how to reduce asthma triggers”; “Controlled asthma improves health and well-being” in educational booklets significantly enhanced caregivers’ self-efficacy and response efficacy for asthma management. In addition, other studies with similar intervention content significantly improved the self-efficacy and response efficacy of other caregivers of children with eczema and food allergy [65,66]. In contrast, susceptibility perceptions showed no significant intergroup difference, potentially due to high baseline awareness of genetic risks in families with atopic histories [67]. Saturation in preexisting risk cognition may have limited further elevation. Meanwhile, limited intergroup changes were observed in internal or external rewards, possibly because caregivers inherently viewed disease management as an obligatory responsibility rather than an optional behavior meriting external incentives [68]. Future interventions should be tailored to specific characteristics (eg, family history) of the target population, with adjustments in content of each PMT dimension to achieve a more balanced and comprehensive enhancement in parental motivation.

Our PMT-CBO intervention elicited notable improvements in caregivers’ behavioral intentions but limited behavioral changes, suggesting a time lag between cognition and action. The observed discrepancy between enhanced behavioral intentions and only modest behavioral adjustments may indicate that the effects of cognitive improvements require a longer duration to manifest as observable changes in behavior [69]. To address this, it is recommended that future interventions not only continue to focus on enhancing cognitive mediators but also incorporate strategies from the theory of planned behavior (TPB) to reinforce the intention-behavior link [70]. Integrating TPB principles could help caregivers in developing concrete action plans and setting realistic behavioral goals [71,72]. Moreover, external environmental factors appear to play a moderating role in shaping health behaviors. For instance, limited access to health care resources and the burden of economic costs may further impede the adoption of improved disease management behaviors despite increased intentions [73,74]. Such external constraints emphasize the need for multilevel environmental support strategies and ecological frameworks for health promotion.

### Benefits and Challenges of Integrated Interventions

The multidimensional integrated intervention in this study demonstrated significant synergistic advantages in managing allergic diseases by addressing shared pathophysiological pathways and behavioral mechanisms. Specifically, epithelial barrier dysfunction and Th2-mediated inflammation may be alleviated by our unified interventions, such as instructions of daily moisturizing routines and environmental trigger avoidance, which collectively disrupt the progress of “atopic march.” This aligns with recent evidence showing that early skin barrier enhancement in infants [75-77] or allergen reduction [78-80] can lower IgE sensitization risks and reduce comorbid allergies, supporting the clinical value of holistic approaches over siloed disease management. Furthermore, the cross-disease enhancement of medication adherence may be explained by skill transferability. Caregivers who mastered topical steroid application for eczema could seamlessly adapt these techniques to rhinitis nasal sprays, reinforcing self-efficacy through competency generalization. Positive feedback loops emerged as symptom relief in one condition (eg, reduced eczema pruritus) increased caregivers’ confidence in managing comorbid allergies, creating a “virtuous cycle” of proactive care [81,82]. These findings advocate for standardized clinical protocols integrating barrier maintenance, environmental controls, and skill-based medication training as foundational components of pediatric allergy prevention programs.

Despite these synergies, disease-specific challenges persist, particularly in asthma and food allergy management. The suboptimal asthma control outcomes in this study may reflect the unique temporal and technical demands of this condition—unpredictable exacerbations disrupt caregivers’ threat appraisal consistency, while an improper inhaler technique negates controller medication efficacy. Prior research indicates that 60% to 80% of caregivers commit critical inhaler errors [83,84] and the number decreased significantly after standard training [85,86], underscoring the need for condition-specific reinforcement. Similarly, the unmitigated burden of food allergy management exposes a conflict between uncontrollable social dining risks and hypervigilant parenting behaviors. Caregivers tended to avoid social gatherings (eg, birthday parties) due to allergy anxieties or worries, paradoxically increasing children’s social isolation and loneliness risks [87-89]. To address these disparities, modular intervention add-ons could be developed in the future, such as gamified inhaler trainers with real-time artificial intelligence feedback [90,91], or virtual reality simulations of restaurants and food to desensitize families to controlled allergen exposures [92,93]. Embedding these condition-specific modules within the current core PMT framework would preserve integration benefits while accommodating disease heterogeneity—a critical step toward precision and individualized allergy care.

### Implications for Future Pediatric Atopic Interventions

Our findings revealed the multidimensional complexity of pediatric allergy management. On the basis of this PMT trial, we proposed a stepped-care program combining core PMT modules, characteristic or condition-specific plug-ins, and community support systems to balance scalability with personalization in the future. PMT-based core modules would deliver universal behavioral principles (eg, trigger identification, emergency protocols, and adherence improvement), as validated in our study. Meanwhile, characteristic- or condition-specific plug-ins could tailor content to dynamic needs, such as notifying personalized genetic risk feedback, intensifying asthma action plans during pollen seasons [94], or informing food allergy labeling in shopping and dining scenarios [95]. Moreover, merging the PMT with the TPB or other implementation intention techniques (eg, “if-then” planning for asthma exacerbations) may strengthen the intention-behavior pathway [96,97], as shown in children’s oral health programs [98]. To further enhance PMT’s applicability to atopic management, theoretical integrations should also be considered. While the PMT effectively addresses individual-level cognition, its limited emphasis on social and environmental determinants constrains behavior change in real-world settings. Therefore, incorporating constructs from the socio-ecological model may help address these multilevel barriers, such as health care system support (eg, regular allergy testing access) and community or school resources (eg, school allergy-free policies) [99-101].

### Strengths and Limitations

Our study explored the application of PMT to develop integrated caregiver-mediated interventions for children with atopic diseases, novelly considering the progression of the atopic march and comorbidity patterns through a cognitive behavioral framework. We developed multidisciplinary intervention resources (eg, trigger identification and emergency preparedness) to address key and universal challenges in atopic disease management, ultimately improving caregivers’ protection motivation, behavioral intentions, and children’s clinical outcomes (eg, eczema severity reduction and medication adherence). In addition, the replicable and scalable online intervention package was embedded within a WeChat mini-program. The WeChat platform is accessible to >1.3 billion users and has no installation barriers in China, ensuring 24-7 availability and continuity of education [102]. The WeChat group chats further facilitated communication between caregivers, the research team, and health care professionals, enhancing intervention effectiveness by providing timely and tailored support.

There are several limitations in this study. First, this study may have limited generalizability as participants were recruited from urban, educated populations in more-developed eastern China [64,103]. Future research should include more diverse samples from rural or less-developed areas for broader representativeness. Second, evaluation of the study relied on online self-reported questionnaires or scales. While these tools demonstrated good reliability and validity, they may be susceptible to subjective bias. Future studies should integrate objective measures and economic indicators to strengthen validity. Third, the 4-week intervention duration was relatively short, necessitating extended follow-up to evaluate its long-term effects on caregivers’ behaviors and children’s health outcomes. Extended follow-ups were needed to evaluate its long-term effects on caregivers’ behaviors and children’s health outcomes. Fourth, the PMT-CBO group showed slightly lower completion rate than the control group (116/127, 91.3% vs 111/116, 95.7%), possibly due to the intervention’s complexity. Future iterative usability testing and optimized incentive strategies may help enhance adherence and completion rate.

### Conclusions

This study demonstrates that a 4-week PMT-CBO intervention effectively enhances caregivers’ motivation, intentions, and partial behaviors in managing pediatric atopic diseases, while improving children’s medication adherence and alleviating eczema and allergic rhinitis symptoms. By integrating the intervention package into a scalable WeChat-based platform, the intervention achieved high accessibility and engagement (>90%), offering a replicable model for digital allergy education. However, limited effects on asthma control and food allergy–related caregiver burdens highlight the need for condition-specific adaptations, such as artificial intelligence–enhanced inhaler training or food allergen risk simulations. Future work should prioritize socioeconomic diversity, objective outcome measures, and ecological frameworks to bridge intention-behavior gaps and promote equitable, precision allergy care.

## Appendix

Multimedia Appendix 1 The Protection Motivation Theory–based cognitive behavioral online intervention protocol and outline.

Multimedia Appendix 2 Atopic disease protection motivation scale for caregivers of children with atopic disease (revised version).

Multimedia Appendix 3 Comparison of protective motivation scores of caregivers in the Protection Motivation Theory–based cognitive behavioral online intervention (PMT-CBO) group (n=127) and control group (n=116) before and after the intervention.

Multimedia Appendix 4 Comparison of the frequency of prevention and treatment behaviors of caregivers in 2 groups before and after the intervention.

Multimedia Appendix 5 Comparison of medication adherence of children with atopic disease in the Protection Motivation Theory–based cognitive behavioral online (PMT-CBO) group (n=127) and the control group (n=116) before and after the intervention.

Multimedia Appendix 6 Comparison of subjective atopic disease symptom score of children with atopic disease in the 2 groups before and after the intervention.

## Abbreviations

AR-SRS: Allergic Rhinitis Symptom Rating Scale
C-ACT: Childhood Asthma Control Test
FAQL-PB: Food Allergy Quality of Life–Parental Burden
GEE: generalized estimating equation
GMAS: General Medication Adherence Scale
PMT: protection motivation theory
PMT-CBO: Protection Motivation Theory–based cognitive behavioral online
POEM: Patient-Oriented Eczema Measure
TPB: theory of planned behavior

## Data Availability

The datasets generated or analyzed during this study are not publicly available due to ethical restrictions but are available from the corresponding author upon reasonable request.
