# Supplementary material for: Examining the Effects of the Protection Motivation Theory–Based Online Intervention on Improving the Cognitive Behavioral Outcomes of Caregivers of Children With Atopic Diseases: Quasi-Experimental Study
Source: J Med Internet Res. 2025 May 13;27:e72925. doi: 10.2196/72925 (PMC12117277; doi:10.2196/72925)
Supplement: Multimedia Appendix 1 [file jmir_v27i1e72925_app1.docx]

**Multimedia Appendix 1.** The Protection Motivation Theory–based cognitive behavioral online intervention protocol and outline.

| **Dissemination Plan** | | **Intervention Pathways** | | | | | | | |
| --- | --- | --- | --- | --- | --- | --- | --- | --- | --- |
|  |  | **Week 1** | | **Week 2** | | **Week 3** | | **Week 4** | |
| **Dimensions** | **Educational content** | **Images**  **/Text** | **Video** | **Images**  **/Text** | **Lecture** | **Video** | **Images**  **/Text** | **Images**  **/Text** | **Video** |
| Susceptibility | 1. What are atopic diseases? What are the typical clinical manifestations of atopic diseases? | √ |  |  |  |  |  |  |  |
|  | 2. What is the atopic march of atopic diseases? | √ |  |  |  |  |  |  |  |
|  | 3. What is the prevalence of atopic diseases in China? | √ |  |  |  |  |  |  |  |
|  | 4. Are atopic diseases hereditary? What kind of children are prone to atopic diseases? | √ | √ |  |  |  |  |  |  |
|  | 5. What environmental factors can trigger the onset of atopic diseases (hygiene hypothesis)? | √ | √ |  |  |  |  |  |  |
| Severity | 1. What behavioral factors can lead to the recurrence of atopic diseases? | √ |  |  |  |  |  |  |  |
|  | 2. What factors can induce severe allergic reactions? | √ | √ |  |  |  |  |  |  |
|  | 3. What complications can arise from not actively treating atopic diseases? | √ |  |  |  |  |  |  |  |
|  | 4. How can caregivers identify and urgently manage them? | √ | √ |  |  |  |  |  |  |
| Internal or external rewards | 1. How to verify the scientific validity of atopic disease prevention and treatment information on the internet? |  |  | √ |  |  |  |  |  |
|  | 2. Are probiotics promoted on the internet really effective in preventing and treating atopic diseases? |  |  | √ |  |  |  |  |  |
|  | 3. Misconceptions in prevention and treatment behavior lecture - Misconception one: Basic cognitive misconceptions about atopic diseases. |  |  |  | √ |  |  |  |  |
|  | 4. Misconceptions in prevention and treatment behavior lecture - Misconception two: Misconceptions about allergen avoidance and environmental control. |  |  |  | √ |  |  |  |  |
|  | 5. Misconceptions in prevention and treatment behavior lecture - Misconception three: Misconceptions about the use of antiallergic drugs. |  |  |  | √ |  |  |  |  |
|  | 6. Misconceptions in prevention and treatment behavior lecture - Misconception four: Misconceptions about medical treatment and referral. |  |  |  | √ |  |  |  |  |
|  | 7. Misconceptions in prevention and treatment behavior lecture - Misconception five: Misconceptions about neglecting daily monitoring. |  |  |  | √ |  |  |  |  |
|  | 8. Misconceptions in prevention and treatment behavior lecture - Misconception six: Misconceptions about daily care. |  |  |  | √ |  |  |  |  |
| Self-efficacy | 1. What are the common allergens? How to avoid them? |  |  |  |  | √ | √ |  |  |
|  | 2. What are tips for using antiallergic drugs according to medical prescription? |  |  |  |  | √ | √ |  |  |
|  | 3. How can children with atopic diseases arrange their diet reasonably? |  |  |  |  | √ | √ |  |  |
|  | 4. What sports can children with atopic conditions do? What are the precautions? |  |  |  |  | √ | √ |  |  |
|  | 5. How can families of children with atopic diseases coexist with the disease? |  |  |  |  | √ | √ |  |  |
|  | 6. Correct steps and precautions for nasal irrigation. |  |  |  |  | √ | √ |  |  |
|  | 7. Correct steps and precautions for nebulization. |  |  |  |  | √ | √ |  |  |
|  | 8. Correct steps and precautions for bathing and skin care. |  |  |  |  | √ | √ |  |  |
| Response efficacy | 1. How to scientifically understand allergen testing? |  |  |  |  | √ | √ |  |  |
|  | 2. Why does the treatment of atopic diseases need to be long-term? |  |  |  |  | √ | √ |  |  |
|  | 3. What are the short-term and long-term benefits of actively preventing and treating atopic diseases for children? |  |  |  |  | √ | √ |  |  |
|  | 4. What are the benefits of actively preventing and treating atopic diseases for families? |  |  |  |  | √ | √ |  |  |
|  | 5. How to distinguish atopic rhinitis and asthma from the common cold? |  |  |  |  |  | √ |  |  |
|  | 6. How to distinguish eczema from common skin diseases such as chickenpox? |  |  |  |  |  | √ |  |  |
|  | 7. How to choose the right hospital and department? |  |  |  |  |  | √ |  |  |
|  | 8. What are the benefits of caregivers supervising their children’s medication and daily care? |  |  |  |  |  | √ |  |  |
| Response cost | 1. Why does the child’s condition not improve after treatment? |  |  |  |  |  |  | √ |  |
|  | 2. Why should an atopic diary be kept for children with atopic disease? How to assess the child’s condition? |  |  |  |  |  |  |  | √ |
|  | 3. Can atopic diseases be cured? |  |  |  |  |  |  | √ |  |
|  | 4. Should children with atopic diseases avoid certain foods? |  |  |  |  |  |  | √ |  |
|  | 5. Can children with atopic diseases receive vaccinations? For example, COVID-19, influenza, and HPV vaccines. |  |  |  |  |  |  | √ |  |
|  | 6. How to reduce unnecessary expenses related to atopic diseases? |  |  |  |  |  |  | √ |  |
|  | 7. What is the safety of hormone treatment for atopic diseases, and why can’t hormones be stopped immediately after use? |  |  |  |  |  |  | √ |  |
|  | 8. Why is allergen avoidance of great significance in the prevention and treatment of atopic diseases? |  |  |  |  |  |  | √ |  |
