# Supplementary material for: Examining the Effects of the Protection Motivation Theory–Based Online Intervention on Improving the Cognitive Behavioral Outcomes of Caregivers of Children With Atopic Diseases: Quasi-Experimental Study
Source: J Med Internet Res. 2025 May 13;27:e72925. doi: 10.2196/72925 (PMC12117277; doi:10.2196/72925)
Supplement: Multimedia Appendix 2 [file jmir_v27i1e72925_app2.docx]

**Multimedia Appendix 2.** Atopic disease protection motivation scale for caregivers of children with atopic disease (revised version).

| Dimensions | Items | Strongly disagree | Disagree | Neutral | Agree | Strongly agree |
| --- | --- | --- | --- | --- | --- | --- |
|  |  |  |  |  |  |  |
| Susceptibility | 1. My child has risk factors for atopic diseases. (eg, genetics, environmental issues, infections, etc.) | 1 | 2 | 3 | 4 | 5 |
|  | 2. Compared to other children, my child is more susceptible to allergic diseases. | 1 | 2 | 3 | 4 | 5 |
|  | 3. If my child already has an allergic disease, he is more likely to have other allergic diseases in the future. | 1 | 2 | 3 | 4 | 5 |
| Severity | 4. Atopic diseases can have many adverse effects on my children. | 1 | 2 | 3 | 4 | 5 |
|  | 5. Atopic disease attacks can affect my child's daily life. (such as attendance, social activities, sleep, etc.) | 1 | 2 | 3 | 4 | 5 |
|  | 6. Inadequate prevention and treatment of atopic diseases can lead to other complications. (such as adenoid hypertrophy, sinusitis, otitis media, etc.) | 1 | 2 | 3 | 4 | 5 |
| Self-efficacy | 7. I believe I am capable of preventing and controlling my child's allergic diseases. | 1 | 2 | 3 | 4 | 5 |
|  | 8. I am proactive in searching for knowledge related to my child’s allergic diseases. | 1 | 2 | 3 | 4 | 5 |
|  | 9. Implementing preventive and treatment behaviors for my child's atopic diseases is easy for me. | 1 | 2 | 3 | 4 | 5 |
| Response efficacy | 10. Actively managing my child's allergic diseases reflects my sense of responsibility. | 1 | 2 | 3 | 4 | 5 |
|  | 11. Proactive preventive behavior can reduce the onset of atopic diseases in children. | 1 | 2 | 3 | 4 | 5 |
|  | 12. Daily attention to my child's allergy symptoms helps to reduce the incidence of atopic diseases in my child. | 1 | 2 | 3 | 4 | 5 |
|  | 13. Taking actions to prevent and control my child’s allergic diseases can promote his/her healthy growth. | 1 | 2 | 3 | 4 | 5 |
|  | 14. My child can benefit from my active prevention and control of allergic diseases. | 1 | 2 | 3 | 4 | 5 |
|  | 15. Avoiding allergens helps reduce my child’s attacks of allergic diseases. | 1 | 2 | 3 | 4 | 5 |
|  | 16. Following the doctor’s prescriptions and using medication properly can help reduce my child’s allergic attacks. | 1 | 2 | 3 | 4 | 5 |
| Response costs | 17. I am not sure if certain allergy prevention behaviors are effective. | 1 | 2 | 3 | 4 | 5 |
|  | 18. I am concerned about the side effects of the medication. (such as hormone drugs, etc.) | 1 | 2 | 3 | 4 | 5 |
|  | 19. I will stop the medication or reduce the dosage for my child on my own. | 1 | 2 | 3 | 4 | 5 |
| Internal or external rewards | 20. If I don’t need to prevent my child’s allergic diseases, I can feel a bit more relaxed. | 1 | 2 | 3 | 4 | 5 |
|  | 21. If I don’t need to prevent my child’s allergic diseases, I can have more free time. | 1 | 2 | 3 | 4 | 5 |
|  | 22. If I don’t need to prevent my child’s allergic diseases, I can have more leisure entertainment and social activities. | 1 | 2 | 3 | 4 | 5 |
