# Supplementary material for: Examining the Effects of the Protection Motivation Theory–Based Online Intervention on Improving the Cognitive Behavioral Outcomes of Caregivers of Children With Atopic Diseases: Quasi-Experimental Study
Source: J Med Internet Res. 2025 May 13;27:e72925. doi: 10.2196/72925 (PMC12117277; doi:10.2196/72925)
Supplement: Multimedia Appendix 3 [file jmir_v27i1e72925_app3.docx]

**Multimedia Appendix 3.** Comparison of protective motivation scores of caregivers in the Protection Motivation Theory–based cognitive behavioral online intervention (PMT-CBO) group (n=127) and control group (n=116) before and after the intervention.

| Dimensions | Group (n/N, %) | Phase | PMT scores, median (IQR) | Score difference, median (IQR) | *Z* value | Intragroup *P* value^a^ | *Z* value | Intergroup *P* value^b^ |
| --- | --- | --- | --- | --- | --- | --- | --- | --- |
| Susceptibility | PMT-CBO^c^ (127/243, 52.3%) |  |  | 0 (0-8.33) | -4.949 | **<.001** | -1.321 | .19 |
|  |  | Preintervention | 83.33 (66.67-91.67) |  |  |  |  |  |
|  |  | Postintervention | 83.33 (75.00-91.67) |  |  |  |  |  |
|  | Control (116/243, 47.7%) |  |  | 0 (0-8.33) | -3.930 | **<.001** |  |  |
|  |  | Preintervention | 75.00 (75.00-83.33) |  |  |  |  |  |
|  |  | Postintervention | 83.33 (75.00-91.67) |  |  |  |  |  |
| Severity | PMT-CBO (127/243, 52.3%) |  |  | 8.33 (0-16.67) | -8.203 | **<.001** | -5.155 | **<.001** |
|  |  | Preintervention | 75.00 (75.00-83.33) |  |  |  |  |  |
|  |  | Postintervention | 91.67 (83.33-100) |  |  |  |  |  |
|  | Control (116/243, 47.7%) |  |  | 0 (0-8.33) | -2.233 | **.03** |  |  |
|  |  | Preintervention | 75.00 (75.00-83.33) |  |  |  |  |  |
|  |  | Postintervention | 83.33 (75.00-83.33) |  |  |  |  |  |
| Self-efficacy | PMT-CBO (127/243, 52.3%) |  |  | 8.33 (0-16.67) | -8.305 | **<.001** | -6.891 | **<.001** |
|  |  | Preintervention | 58.33 (50.00-66.67) |  |  |  |  |  |
|  |  | Postintervention | 75.00 (58.33-83.33) |  |  |  |  |  |
|  | Control (116/243, 47.7%) |  |  | 0 (0-8.33) | -4.724 | **<.001** |  |  |
|  |  | Preintervention | 58.00 (50.00-66.67) |  |  |  |  |  |
|  |  | Postintervention | 58.33 (50.00-66.67) |  |  |  |  |  |
| Response efficacy | PMT-CBO (127/243, 52.3%) |  |  | 3.57 (0-10.71) | -7.852 | **<.001** | -3.342 | **<.001** |
|  |  | Preintervention | 78.57 (75.00-89.29) |  |  |  |  |  |
|  |  | Postintervention | 85.71 (78.57-96.43) |  |  |  |  |  |
|  | Control (116/243, 47.7%) |  |  | 3.57 (0-3.58) | -5.498 | **<.001** |  |  |
|  |  | Preintervention | 82.14 (75.00-85.71) |  |  |  |  |  |
|  |  | Postintervention | 82.14 (78.57-89.29) |  |  |  |  |  |
| Response cost | PMT-CBO (127/243, 52.3%) |  |  | 8.33 (0-16.67) | -7.326 | **<.001** | -5.963 | **<.001** |
|  |  | Preintervention | 41.67 (33.33-50.00) |  |  |  |  |  |
|  |  | Postintervention | 50.00 (41.67-58.33) |  |  |  |  |  |
|  | Control (116/243, 47.7%) |  |  | 0 (-8.33-8.33) | -0.723 | .47 |  |  |
|  |  | Preintervention | 41.67 (33.33-41.67) |  |  |  |  |  |
|  |  | Postintervention | 41.67 (33.33-41.67) |  |  |  |  |  |
| Internal or external rewards | PMT-CBO (127/243, 52.3%) |  |  | 8.33 (0-16.66) | -5.765 | **<.001** | -0.989 | .32 |
|  |  | Preintervention | 50.00 (25.00-58.33) |  |  |  |  |  |
|  |  | Postintervention | 50.00 (41.67-58.33) |  |  |  |  |  |
|  | Control (116/243, 47.7%) |  |  | 8.33 (-6.25-16.67) | -3.138 | **.002** |  |  |
|  |  | Preintervention | 41.67 (33.33-50.00) |  |  |  |  |  |
|  |  | Postintervention | 50.00 (41.67-50.00) |  |  |  |  |  |
| Overall PMT score | PMT-CBO (127/243, 52.3%) |  |  | 5.68 (3.41-12.50) | -9.024 | **<.001** | -6.289 | **<.001** |
|  |  | Preintervention | 65.91 (60.23-72.73) |  |  |  |  |  |
|  |  | Postintervention | 76.14 (69.32-80.68) |  |  |  |  |  |
|  | Control (116/243, 47.7%) |  |  | 2.27 (0-5.68) | -6.090 | **<.001** |  |  |
|  |  | Preintervention | 65.91 (62.50-68.18) |  |  |  |  |  |
|  |  | Postintervention | 69.32 (64.77-71.59) |  |  |  |  |  |

^a^Wilcoxon signed-rank test.

^b^Mann-Whitney *U* test.

^c^PMT-CBO: Protection Motivation Theory–based cognitive behavioral online intervention.
