# Supplementary material for: Examining the Effects of the Protection Motivation Theory–Based Online Intervention on Improving the Cognitive Behavioral Outcomes of Caregivers of Children With Atopic Diseases: Quasi-Experimental Study
Source: J Med Internet Res. 2025 May 13;27:e72925. doi: 10.2196/72925 (PMC12117277; doi:10.2196/72925)
Supplement: Multimedia Appendix 4 [file jmir_v27i1e72925_app4.docx]

**Multimedia Appendix 4.** Comparison of the frequency of prevention and treatment behaviors of caregivers in 2 groups before and after the intervention.

| Group | n (%) | Preintervention^a^ | | | | | Postintervention^a^ | | | | | *Z* value | Intragroup *P* value^b^ |
| --- | --- | --- | --- | --- | --- | --- | --- | --- | --- | --- | --- | --- | --- |
|  |  | 1 | 2 | 3 | 4 | 5 | 1 | 2 | 3 | 4 | 5 |  |  |
| Adopt general preventive measures for atopic diseases. | | | | | | | | | | | |  |  |
| PMT-CBO^c^ | 127 (52.3%) | 8 | 57 | 54 | 7 | 1 | 12 | 62 | 50 | 3 | 0 | -1.701 | .09 |
| Control | 116 (47.7%) | 6 | 51 | 41 | 17 | 1 | 8 | 55 | 45 | 8 | 0 | -1.564 | .12 |
| *Z* value |  | -0.991 | | | | | -1.016 | | | | |  |  |
| Intergroup *P* value^d^ |  | .32 | | | | | .31 | | | | |  |  |
| Monitor the child’s condition regularly. | | | | | | | | | | | |  |  |
| PMT-CBO | 127 (52.3%) | 12 | 60 | 25 | 16 | 14 | 16 | 62 | 23 | 20 | 6 | -1.110 | .27 |
| Control | 116 (47.7%) | 7 | 49 | 27 | 17 | 16 | 13 | 52 | 24 | 17 | 10 | -1.718 | .09 |
| *Z* value |  | -1.391 | | | | | -0.888 | | | | |  |  |
| Intergroup *P* value |  | .16 | | | | | .37 | | | | |  |  |
| Receive allergy-related health education. | | | | | | | | | | | |  |  |
| PMT-CBO | 127 (52.3%) | 2 | 37 | 44 | 24 | 20 | 10 | 60 | 31 | 19 | 7 | -4.217 | **<.001** |
| Control | 116 (47.7%) | 3 | 31 | 39 | 20 | 23 | 9 | 39 | 31 | 26 | 11 | -2.074 | **.04** |
| *Z* value |  | -0.460 | | | | | -2.149 | | | | |  |  |
| Intergroup *P* value |  | .65 | | | | | **.03** | | | | |  |  |
| Child was sick due to exposure to allergens | | | | | | | | | | | |  |  |
| PMT-CBO | 127 (52.3%) | 5 | 34 | 56 | 26 | 8 | 3 | 25 | 46 | 44 | 9 | -2.376 | **.02** |
| Control | 116 (47.7%) | 8 | 39 | 55 | 11 | 3 | 6 | 37 | 40 | 21 | 12 | -2.162 | **.03** |
| *Z* value |  | -2.429 | | | | | -2.396 | | | | |  |  |
| Intergroup *P* value |  | **.02** | | | | | **.02** | | | | |  |  |
| Child attends follow-up appointments regularly as doctors required. | | | | | | | | | | | |  |  |
| PMT-CBO | 127 (52.3%) | 12 | 57 | 48 | 5 | 5 | 16 | 72 | 33 | 2 | 4 | -2.371 | **.02** |
| Control | 116 (47.7%) | 9 | 47 | 49 | 11 | 0 | 14 | 54 | 35 | 7 | 6 | -0.629 | .53 |
| *Z* value |  | -0.914 | | | | | -1.629 | | | | |  |  |
| Intergroup *P* value |  | .36 | | | | | .10 | | | | |  |  |
| Child adheres to prescribed medication regimens. | | | | | | | | | | | |  |  |
| PMT-CBO | 127 (52.3%) | 13 | 73 | 38 | 2 | 1 | 22 | 80 | 19 | 6 | 0 | -2.077 | **.04** |
| Control | 116 (47.7%) | 11 | 62 | 43 | 0 | 0 | 16 | 67 | 30 | 3 | 0 | -1.191 | .23 |
| *Z* value |  | -0.595 | | | | | -1.421 | | | | |  |  |
| Intergroup *P* value |  | .55 | | | | | .16 | | | | |  |  |

^a^The items were as follows: 1, always; 2, often; 3, sometimes; 4, occasionally; and 5, never.

^b^Wilcoxon signed-rank test.

^c^PMT-CBO: Protection Motivation Theory–based cognitive behavioral online intervention.

^d^Mann-Whitney *U* test.
