# Supplementary material for: Examining the Effects of the Protection Motivation Theory–Based Online Intervention on Improving the Cognitive Behavioral Outcomes of Caregivers of Children With Atopic Diseases: Quasi-Experimental Study
Source: J Med Internet Res. 2025 May 13;27:e72925. doi: 10.2196/72925 (PMC12117277; doi:10.2196/72925)
Supplement: Multimedia Appendix 5 [file jmir_v27i1e72925_app5.docx]

**Multimedia Appendix 5.** Comparison of medication adherence of children with atopic disease in the Protection Motivation Theory–based cognitive behavioral online (PMT-CBO) group (n=127) and the control group (n=116) before and after the intervention.

| **Group** (n/N, %) | **Phase** | **GMAS^a^ score, median (IQR)** | **Score difference, median (IQR)** | ***Z* value** | **Intragroup*P* value^b^** | ***Z* value** | **Intergroup**  ***P* value^c^** |
| --- | --- | --- | --- | --- | --- | --- | --- |
| PMT-CBO^d^ (127/243, 52.3%) |  |  | 1.00 (0-2.00) | -7.970 | **<.001** | -4.457 | **<.001** |
|  | Preintervention | 25.00 (24.00-27.00) |  |  |  |  |  |
|  | Postintervention | 26.00 (25.00-29.00) |  |  |  |  |  |
| Control (116/243, 47.7%) |  |  | 0 (-1.00-2.00) | -1.673 | .09 |  |  |
|  | Preintervention | 25.00 (24.00-26.00) |  |  |  |  |  |
|  | Postintervention | 25.00 (24.00-27.00) |  |  |  |  |  |

^a^GMAS: General Medication Adherence Scale.

^b^Wilcoxon signed-rank test.

^c^Mann-Whitney *U* test.

^d^PMT-CBO: Protection Motivation Theory–based cognitive behavioral online intervention.
