# Supplementary material for: Examining the Effects of the Protection Motivation Theory–Based Online Intervention on Improving the Cognitive Behavioral Outcomes of Caregivers of Children With Atopic Diseases: Quasi-Experimental Study
Source: J Med Internet Res. 2025 May 13;27:e72925. doi: 10.2196/72925 (PMC12117277; doi:10.2196/72925)
Supplement: Multimedia Appendix 6 [file jmir_v27i1e72925_app6.docx]

**Multimedia Appendix 6.** Comparison of subjective atopic disease symptom score of children with atopic disease in the 2 groups before and after the intervention.

| **Atopic disease symptom score scales** | **Group (n/N ,%)** | **Phase** | **Symptom score, median (IQR) / mean (SD)** | **Score difference, median (IQR) / mean (SD)** | ***t/Z* value** | **Intragroup *P* value** | ***Z* value** | **Intergroup**  ***P*^a^ value** |
| --- | --- | --- | --- | --- | --- | --- | --- | --- |
| POEM^b^ | PMT-CBO^c^ (21/39, 54%) |  |  | 2.00 (2.00-3.00)^d^ | -4.070 | **<.001^e^** | -3.112 | **.002** |
|  |  | Preintervention | 7.00 (4.00-12.00)^d^ |  |  |  |  |  |
|  |  | Postintervention | 5.24 (3.66)^f^ |  |  |  |  |  |
|  | Control (18/39, 46%) |  |  | 0.89 (1.81)^f^ | -1.861 | .06^e^ |  |  |
|  |  | Preintervention | 6.50 (5.00-9.00)^d^ |  |  |  |  |  |
|  |  | Postintervention | 5.50 (5.00-7.25)^d^ |  |  |  |  |  |
| FAQL-PB^g^ | PMT-CBO (25/59, 42%) |  |  | 0.23 (0.17-0.32)^d^ | -4.378 | **<.001^e^** | -1.693 | .09 |
|  |  | Preintervention | 2.29 (1.94-2.41)^d^ |  |  |  |  |  |
|  |  | Postintervention | 1.94 (1.68-2.18)^d^ |  |  |  |  |  |
|  | Control (34/59, 58%) |  |  | 0.18 (0.06-0.29)^d^ | -4.307 | **<.001^e^** |  |  |
|  |  | Preintervention | 1.94 (1.81-2.29)^d^ |  |  |  |  |  |
|  |  | Postintervention | 1.80 (0.48)^f^ |  |  |  |  |  |
| AR-SRS^h^ | PMT-CBO (103/195, 52.8%) |  |  | 1.00 (0-2.00)^d^ | -3.140 | **.002^e^** | -3.277 | **<.001** |
|  |  | Preintervention | 4.00 (3.00-5.00)^d^ |  |  |  |  |  |
|  |  | Postintervention | 3.00 (2.00-4.00)^d^ |  |  |  |  |  |
|  | Control (92/195, 47.2%) |  |  | 0 (0-1.00)^d^ | -1.734 | .08^e^ |  |  |
|  |  | Preintervention | 4.00 (3.00-4.00)^d^ |  |  |  |  |  |
|  |  | Postintervention | 4.00 (3.00-4.00)^d^ |  |  |  |  |  |
| C-ACT^i^ | PMT-CBO (47/69, 68%) |  |  | 1.00 (0-2.00)^d^ | -5.582 | **<.001^j^** | -.830 | .41 |
|  |  | Preintervention | 19.38 (3.79)^f^ |  |  |  |  |  |
|  |  | Postintervention | 21.15 (3.28)^f^ |  |  |  |  |  |
|  | Control (22/69, 32%) |  |  | 1.05 (1.40)^f^ | -3.440 | **.003^j^** |  |  |
|  |  | Preintervention | 19.62 (2.75)^f^ |  |  |  |  |  |
|  |  | Postintervention | 20.67 (2.24)^f^ |  |  |  |  |  |

^a^Mann-Whitney *U* test.

^b^POEM: Patient-Oriented Eczema Measure.

^c^PMT-CBO: Protection Motivation Theory–based cognitive behavioral online intervention.

^d^The values are median (IQR).

^e^Wilcoxon signed-rank test.

^f^The values are mean (SD).

^g^FAQL-PB: Food Allergy Quality of Life–Parental Burden.

^h^AR-SRS: Allergic Rhinitis Symptom Rating Scale.

^i^C-ACT: Childhood Asthma Control Test.

^j^Paired *t* test.
